# NF-κB pathways are involved in M1 polarization of RAW 264.7 macrophage by polyporus polysaccharide in the tumor microenvironment

**DOI:** 10.1371/journal.pone.0188317

**Published:** 2017-11-20

**Authors:** Chun-Ping Liu, Xian Zhang, Qing-Long Tan, Wen-Xing Xu, Chang-Yuan Zhou, Min Luo, Xiong Li, Run-Yue Huang, Xing Zeng

**Affiliations:** 1 Department of Integrated Chinese medicine immunization and Section Rheumatology Research, The Second Affiliated Hospital, Guangzhou University of Chinese Medicine (Guangdong Provincial Hospital of Chinese Medicine), Guangzhou, China; 2 Discipline of Integrated Chinese and Western Medicine in Guangzhou University of Chinese medicine, Guangzhou, China; 3 Guangdong Provincial Key Laboratory of Clinical Research on Traditional Chinese Medicine Syndrome, Guangzhou, China; Okayama University, JAPAN

## Abstract

Bladder cancer is one of the most malignant tumors closely associated with macrophages. Polyporus polysaccharide (PPS) has shown excellent efficacy in treating bladder cancer with minimal side effects. However, the molecular mechanisms underlying the effects of PPS in inhibiting bladder cancer remain unclear. In this study, we used macrophages cultured alone or with T24 human bladder cancer cell culture supernatant as study models. We found that PPS enhanced the activities of IFN-γ-stimulated RAW 264.7 macrophages, as shown by the release of inducible nitric oxide synthase (INOS), secretion of tumor necrosis factor (TNF)-α and interleukin (IL)-6, phagocytosis activity, as well as expression of M1 phenotype indicators, such as CD40, CD284 and CD86. PPS acted upstream in activation cascade of nuclear factor (NF)-κB signaling pathways by interfering with IκB phosphorylation. In addition, PPS regulated NF-κB (P65) signaling by interfering with Toll-like receptor (TLR)-4, INOS and cyclooxygenase (COX)-2. Our results indicate that PPS activates macrophages through TLR4/NF-κB signaling pathways.

## Introduction

Bladder cancer is one of the most commonly diagnosed diseases and is characterized by a high recurrence rate [1,2]. Intravesical instillations of bacillus Calmette-Guérin (BCG) are the most effective treatment to prevent recurrence and progression, whereas approximately 30–40% of patients fail to respond to BCG immunotherapy [3]. It is found that tumor-associated macrophages (TAMs) are one of the most important promoters of tumor progression, angiogenesis and metastasis[4].

As a medicinal fungus, Polyporus has been frequently used in Chinese Medicine to treat edema and various kidney diseases, and importantly, no side effects or toxicities have yet been reported [5]. Polyporus polysaccharide (PPS) is extracted from polyporus and has been well documented to exert anti-tumor, antioxidant and immunoregulatory effects.

Our previous experiments were carried out to identify the toxicity of PPS in animal model of bladder cancer. Mortality in animals treated with the combination of PPS and BCG is lower than animals treated with BCG alone. In addition, compared with animals treated with the combination of polyporus decoction and BCG, the mortality of animals treated by PPS combined with BCG is lower as well [6,7]. PPS has been addressed to be highly effective in inhibiting bladder carcinogenesis in rats [6]. In addition, many studies have reported that PPS can boost nonspecific immunity by stimulating natural killer, T and B cells, and macrophages [8–11].

Cells produced by the differentiation of monocytes in tissues have long been recognized as heterogeneous cells that can express different functional programs in response to microenvironmental signals [12]. M1 and M2 macrophages are two classic activation states that have different functions. M1 macrophages defend against external pathogens by releasing cytotoxic and inflammatory mediators, such as nitric oxide (NO), inducible nitric oxide synthase (INOS), tumor necrosis factor (TNF)-α, interleukin (IL)-6 and IL-1β, while M2 macrophages typically produce IL-10 and tumor growth factor (TGF)-β [12,13]. In the tumor microenvironment, macrophages may become TAMs, which are considered to have the M2 phenotype and can enhance tumor progression, thereby leading to a poor prognosis [14]. In the previous study, we found that PPS could suppress the growth of tumor cell through TLR4/NF-κB pathway [15], and it could enhance the secretion of NO in vitro [16]. Since INOS, COX-2, and, TNF-alpha, and, IL-6 are important factors in the NF-κB pathway [12,13], we detected whether PPS could enhance immunostimulatory activities in the tumor microenvironment through regulating these factors and pathways.

Therefore, in our study, we determined whether PPS could regulate macrophage polarization in the microenvironment of bladder cancer and thereby exerting anti-cancer effects through TLR4/NF-κB pathway.

## Materials and methods

### Reagents

PPS (purity, 95%) was purchased from Zelang Nanjing Pharmaceutical Co., Ltd. (Nanjing, Jiangsu, China) and was dissolved in cell culture medium. IFN-γ was purchased from PeproTech (Rocky Hill, NJ, USA). Radio-immunoprecipitation Assay (RIPA) Lysis Buffer and antibodies used in Western blot analysis, including anti-phospho-P65-NF-κB, anti- P65-NF-κB, anti-phospho-IκB, anti-IκB, anti-INOS, anti-cyclooxygenase (COX)-2, anti-IL-6, anti-TNF-α, and anti-GAPDH were purchased from Cell Signaling Technologies (Pickering, ON, CAN), and secondary antibodies were purchased from Sigma (Sigma, St. Louis, MO, USA). QNZ (purity, 99.09%) was purchased from Selleck. RAW 264.7 macrophages and T24 cells were purchased from American Type Culture Collection (Rockville, MD, USA). Dulbecco's Modified Eagle's Medium (DMEM), fetal bovine serum (FBS) and penicillin/streptomycin solution were purchased from HyClone. A Bicinchoninic Acid (BCA) Protein Assay Kit was obtained from Thermo (Beijing, China). TRIzol was obtained from Takara Bio Inc. (Otsu, Shiga, Japan). An Enhanced Chemiluminescence (ECL) Solution Kit was purchased from Millipore (Billerica, MA, USA). Primers for real-time polymerase chain reaction (RT-PCR) were obtained from Invitrogen (Carlsbad, CA, USA). Monoclonal allophycocyanin (APC)-conjugated anti-CD40, PerCP-Cy^™^ 7-conjugated anti-CD16/32, phycoerythrin (PE)-conjugated anti-CD86 and fluorescein isothiocyanate (FITC)-conjugated anti-mouse CD284 antibodies and Cytometric Bead Array (CBA) Kits were purchased from BD Systems (BD Biosciences, USA).

### RAW 264.7 and T24 cell culture

All cells were maintained in DMEM supplemented with 10% heat-inactivated FBS, 10,000 U/ml penicillin and 10,000 μg/ml streptomycin at 37°C in an incubator with a humidified atmosphere and 5% CO_2_.

### Collection of T24 cell culture supernatant

T24 cells were independently seeded in 10-cm culture dishes at 3×10^6^ cells with 8 mL of medium and cultured for 48 h. Then, the medium was replaced with fresh medium and incubated for 24 h. The culture supernatant was then collected and stored at -80°C.

### RAW 264.7 cell treatment with T24 cell culture supernatant

RAW 264.7 macrophages were seeded onto 12-well culture plates at 5×10^5^ cells per 1 mL or 6-well culture plates at 1×10^6^ cells per 2 mL of culture medium and cultured for 24 h. Subsequently, the cells were incubated with 50% fresh medium or T24 cell culture supernatant for 3 h. Then, the cells were treated with IFN-γ (100 ng/mL) for 3 h. Finally, the IFN-γ-stimulated macrophages were treated with PPS (250 μg/mL) for 8 h, 12 h or 24 h. Using the RAW 264.7 macrophages treated for 8 h with PPS (250 μg/mL), we assessed the protein levels of phospho-P65-NF-κB, phospho-IκB, INOS and COX-2. Using the RAW 264.7 macrophages treated for 24 h with PPS (250 μg/mL), we evaluated the expression of membrane receptors (CD16/32, CD40, CD86 and CD284), the mRNA expression of IL-6, INOS, and TNF-α, and the uptake activity. Using another group of RAW 264.7 macrophages treated with PPS (250 μg/mL) for 24 h, we replaced the culture medium with fresh medium, cultivated the cells for an additional 48 h, and then evaluated the concentrations of NO, IL-6 and TNF-α in the culture medium.

### Quantitative RT-PCR

Total RNA was extracted from cultured cells using TRIzol reagent, and quantitative RT-PCR was performed using an ABI Prism 7500 Sequence Detection System and PCR Master Mix Reagent. The primers used in this study are described in Table 1. The mRNA levels were normalized to that of GAPDH.

**Table 1:** Primer sequences.

Gene	Sense strand (5’-3’)	Antisense strand (3’-5’)
(IL)-6	TACTCGGCAAACCTAGTGCG	GTGTCCCAACATTCATATTGTCAGT
INOS	CGGCAAACATGACTTCAGGC	GCACATCAAAGCGGCCATAG
(TNF)-α	GTCTTGGCCGAGGACTAAGG	GTCTTGGCCGAGGACTAAGG
GAPDH	GTCTTGGCCGAGGACTAAGG	GTCTTGGCCGAGGACTAAGG

### Flow cytometry

Flow cytometry was used to examine the cell phenotypes. RAW 264.7 cells were seeded onto 12-well culture plates at 5×10^5^ cells per well. After 24 h, we replaced the culture medium with 50% fresh medium or T24 cell culture supernatant for 3 h. Subsequently, the cells were incubated with IFN-γ (100 ng/mL) for 3 h and then treated with PPS (250 μg/mL) for 24 h. Finally, the RAW 264.7 cells were harvested and washed twice with cold phosphate-buffered saline (PBS). The cells were incubated with 5 μL of anti-mouse FITC-conjugated anti-CD284, PE-conjugated anti-CD86, APC-conjugated anti-CD40 or isotype-matched controls for 20 min on ice. Then, the stained cells were suspended in cold buffer and analyzed by flow cytometry (FACSAria with Cell Quest software, Beckman FC500).

### Measurement of NO, TNF-α and IL-6 cytokine production

RAW 264.7 cells (5 × 10^5^ cells/well) were seeded in 12-well plates and cultured for 24 h. The cells were then incubated with 50% fresh medium or T24 cell culture supernatant for 3 h before being treated with IFN-γ (100 ng/mL) for 3 h. Subsequently, the cells were treated with PPS (250 μg/mL) for 24 h. After the treatments, we replaced the culture medium with fresh medium, cultivated the cells for an additional 48 h, and then evaluated the concentrations of NO, IL-6 and TNF-α in the culture medium. Nitrite accumulation in the culture supernatant was measured based on the Griess reaction. In addition, the levels of IL-6 and TNF-α in the RAW 264.7 culture supernatant were assayed using a CBA assay kit according to the manufacturer’s instructions.

### Western blot analysis

RAW 264.7 cells were seeded at 1×10^6^ cells/well in 6-well plates and incubated for 24 h. The cells were incubated with 50% fresh medium or T24 cell culture supernatant for 3 h. Then, the cells were treated with IFN-γ (100 ng/mL) for 3 h. Finally, the cells were treated with PPS (250 μg/mL) for 8 h. After the treatments, RAW 264.7 cells were washed twice with pre-cooled PBS and lysed using RIPA buffer containing proteinase and phospho-protein inhibitors. The lysate was then centrifuged at 12,000 × g for 10 min at 4°C, and then the supernatant was collected. A BCA protein assay kit was used to analyze the protein content of the supernatant. Protein samples were loaded, separated by sodium dodecyl sulfate polyacrylamide gel electrophoresis (SDS-PAGE) and transferred to 0.45 μm polyvinylidene difluoride membranes; Susequently, 0.5% bovine serum albumin in 1×Tris-buffered saline (TBS) containing 0.05% Tween-20 was used to block the membranes for 1 h. Protein samples were then separated by SDS-PAGE on 10% gels. The membranes were treated with primary antibodies at 4°C overnight and then treated with horseradish peroxidase-coupled secondary antibodies for 2 h at 4°C. Last, the membranes were washed with TBST after each antibody-binding reaction. An ECL kit was used to detect each protein for imaging and analysis of the experimental results. GAPDH was used as a protein loading control, and the grey level of proteins were calculated by Images lab and Images J.

### Inhibition of NF-κB using a specific inhibitor

To identify the signal transduction pathways that mediate the effects of PPS on IFN-γ-stimulated macrophage activation, RAW 264.7 cells were pretreated with the NF-κB inhibitor QNZ (50 nM) for 2 h in DMEM or a co-culture microenvironment. This medium was subsequently replaced with medium with or without IFN-γ (100 ng/ml) for 3 h. Then, the cells were treated with PPS (250 μg/mL) for 8 h. After treatment, the cells were harvested and analyzed by Western blot.

### Phagocytosis activity

RAW 264.7 cells (5×10^5^ cells/well) were pre-incubated in 6-well plates in a humidified 37°C, 5% CO_2_ incubator. After 24 h, 50% fresh medium or T24 cell culture supernatant was added to each well, and the cells were incubated at 37°C for 3 h. The cells were incubated with IFN-γ (100 ng/ml) for 3 h and were subsequently treated with PPS (250 μg/mL) for 24 h. After the treatments, the cells were re-suspended in 1 mL of PBS with FITC-labeled *E*. *coli* and incubated at room temperature for 20 min. After being washed three times, the cells were analyzed using FACSCalibur (Becton Dickinson, Erembodegem, Belgium). For the immunofluorescence analysis of PPS-mediated phagocytosis, cells were seeded at the same density into 6-well plates and cultured for 24 h. A solution of FITC-labeled *E*. *coli* was added to the cells, which were then incubated for 30 min at 37°C in a humidified incubator containing 5% CO_2_. After being washed three times with cold PBS and fixed with a 4% paraformaldehyde solution, the cells were imaged by fluorescence microscopy.

### UV-VIS spectroscopy

Glucose (50 mg) was dissolved in a 250-mL volumetric flask with ultrapure water.

The glucose solution (0, 1, 2, 3, 4, or 5 mL) was diluted to 50 mL with ultrapure water. Then, 1 mL of a standard solution was added in the tube, followed by the addition of 1 mL of 5% phenol and 1 mL of sulfuric acid. Ultrapure water was used as a blank. The absorbance was measured at 490 nm. In addition, 25.3 mg of PPS was dissolved in a 25-mL volumetric flask with ultrapure water, and the absorbance was measured at 490 nm.

## Statistical analysis

The original data can be found by our DRYAD DOI (doi:10.5061/dryad.6fh45). All data represent the mean of three independent experiments and GraphPad Prism software was used for the statistical analysis. Statistical significance was analyzed using one-way ANOVA with a multiple comparisons post-test (Bonferroni). Data are presented as the mean ± standard error of the mean (SEM). Differences were considered significant at p<0.05.

## Results

### In vitro RAW 264.7 macrophage activation

**Fig 1:**
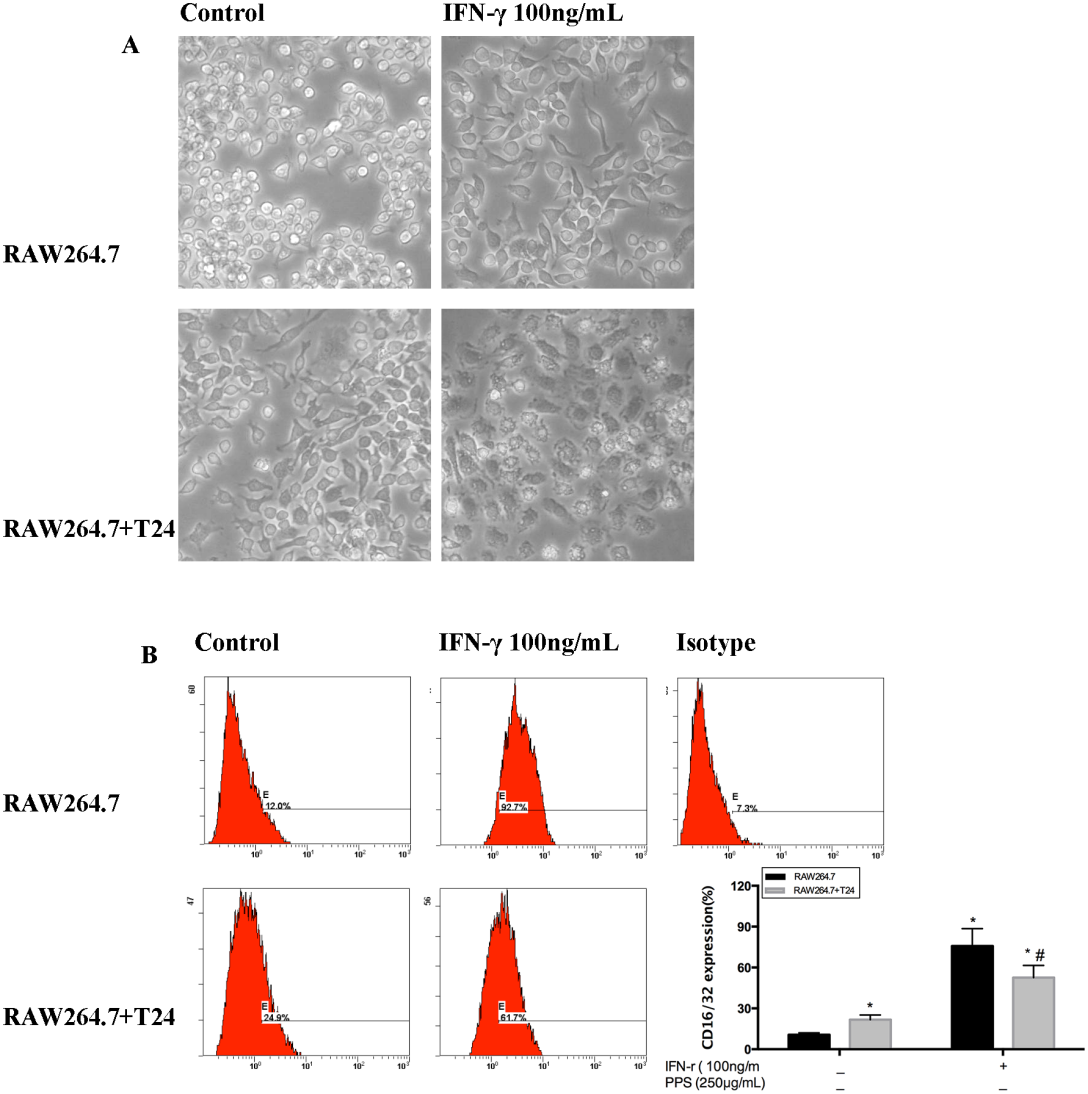
Effect of IFN-γ on the induction of M1-polarized RAW 264.7 cells. Induction of M1-polarized RAW 264.7 cells. RAW 264.7 cells were polarized toward the M1 phenotype by stimulation with 100 ng/mL IFN-γ for 12 h. (A) Microscopy images of IFN-γ-stimulated RAW 264.7 cells in medium or a co-culture microenvironment. (B) Flow cytometry detection of CD16/32, a signature marker of M1 cells. Values are given as the mean ± SD from three independent experiments. Compared with the RAW 264.7 control group, *p<0.05; compared with the RAW 264.7+T24 control group, #p<0.05; compared with the RAW 264.7 IFN-γ group, ▲p<0.05; compared with the RAW 264.7 IFN-γ co-culture group, Δp<0.05.

Fluorescence microscopy was used to image RAW 264.7 cell morphology. We found that IFN-γ induced morphological changes in RAW 264.7 cells, which were differentiated into osteoclast-like cells in the presence of IFN-γ (Fig 1A). Since CD16/32 is a known signature marker of M1 macrophages, a flow cytometric analysis was conducted to examine the CD16/32 cell surface expression. As shown in Fig 1B, CD16/32 was expressed in 92.7% of RAW 264.7 cells exposed to IFN-γ via media. In the co-culture microenvironment, 61.7% of IFN-γ-stimulated macrophages expressed CD16/32, confirming RAW 264.7 cell activation.

### Effect of PPS on RAW 264.7 cell phagocytosis activity

**Fig 2:**
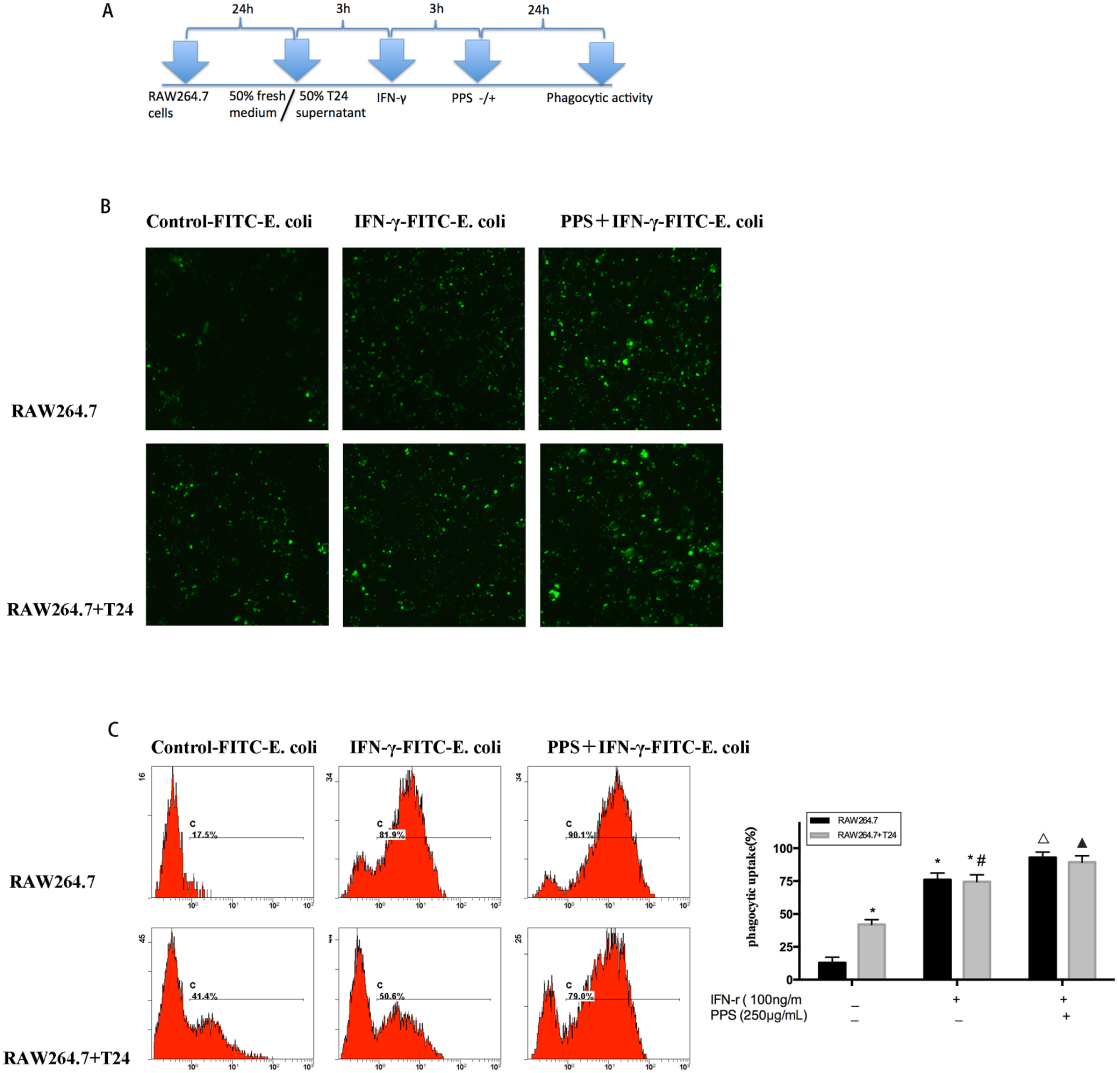
Effect of PPS on the phagocytosis activity of IFN-γ-stimulated RAW 264.7 cells. (A) An illustration of the polarization and PPS treatment of IFN-γ-stimulated RAW 264.7 cells. (B) Phagocytosis activity was analyzed by flow cytometry. (C) Effect of PPS on phagocytosis activity as shown by fluorescence microscopy images. Data represents the mean ± SD of three independent experiments. Compared with the RAW 264.7 control group, *p<0.05; compared with the RAW 264.7+T24 control group, #p<0.05; compared with the RAW 264.7 IFN-γ group, ▲p<0.05; compared with the RAW 264.7 IFN-γ co-culture group, Δp<0.05.

To investigate whether PPS could enhance the phagocytosis activity of IFN-γ-stimulated macrophages, RAW 264.7 cells were pretreated with IFN-γ (100 ng/mL) for 3 h in media or a co-culture microenvironment before being incubated with PPS. Then, the macrophages were incubated with FITC-labeled *E*. *coli*. Immunofluorescence was used to measure uptake efficiency. The fluorescence microscopy results demonstrated that the uptake of FITC-labeled *E*. *coli* was enhanced in IFN-γ-stimulated macrophages treated with PPS (250 μg/mL) (Fig 2B). Flow cytometry further confirmed the data obtained by fluorescence microscopy. In addition, the flow cytometry results showed greater phagocytosis of FITC-labeled *E*. *coli* by IFN-γ-stimulated macrophages treated with PPS than by those not treated with PPS (Fig 2C).

### Effect of PPS on the cytokines NO, IL-6, TNF-α and the signature marker CD86 in IFN-γ-stimulated RAW 264.7 cells

**Fig 3:**
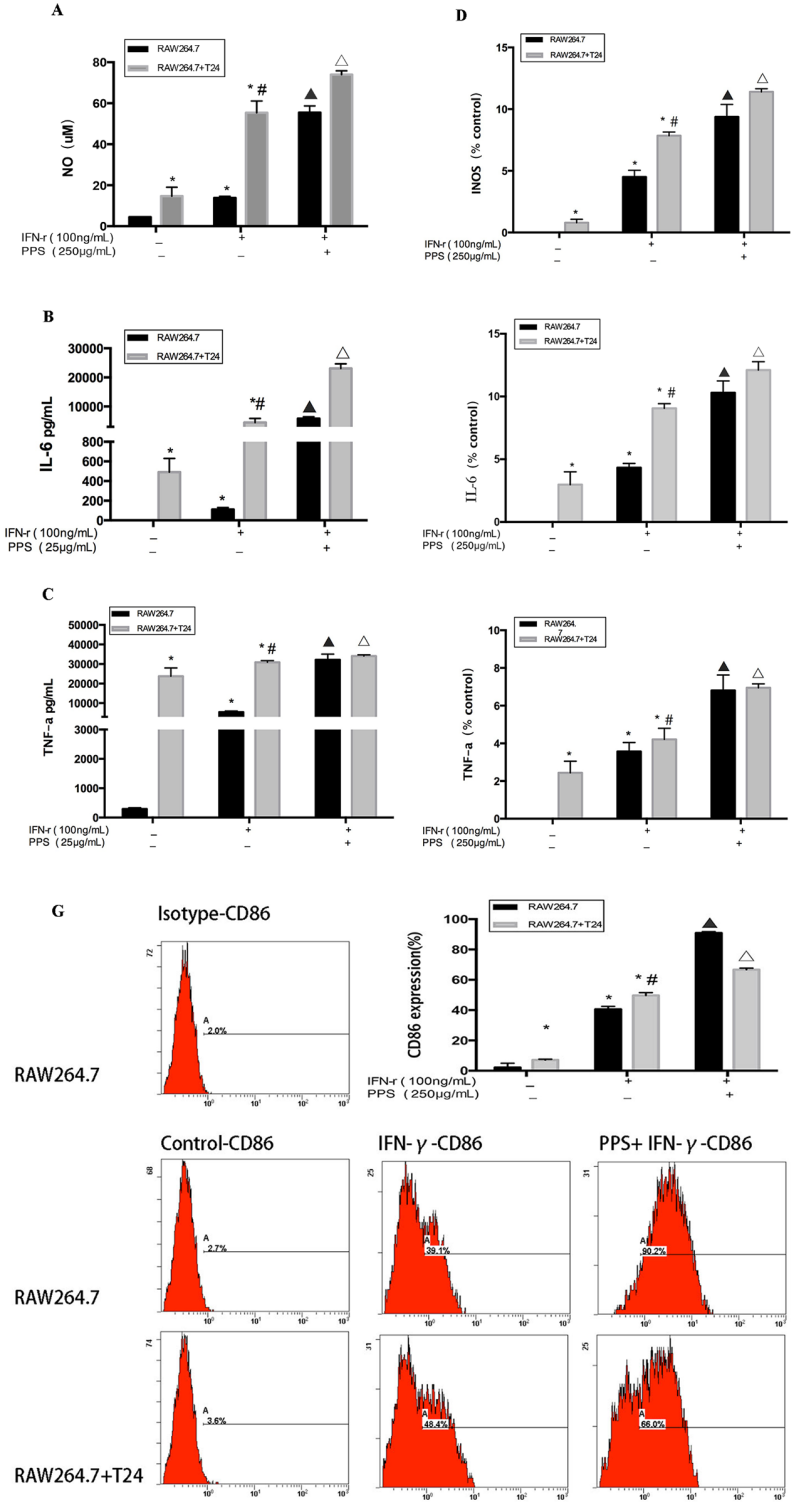
Effect of PPS on the possible mechanism affecting NO, IL-6 and TNF-α secretion in IFN-γ-stimulated RAW 264.7 cells. (A) NO secretion in IFN-γ-stimulated RAW 264.7 cells treated with PPS for 24 h. NO secretion was determined using the Griess Methods. (B-D) The expression of INOS, TNF-α, and IL-6 mRNA in IFN-γ-stimulated RAW 264.7 cells treated with PPS for 24 h, followed by measuring the mRNA levels by qRT-PCR. (E-F) The secretion of TNF-α and IL-6 in IFN-γ-stimulated RAW 264.7 cells treated with PPS for 24 h. Afterwards, the secretion of TNF-α and IL-6 were determined using the Method of CBA. (G) The expression of CD86 in IFN-γ-stimulated RAW 264.7 cells treated with PPS for 24 h. The expression of CD86 were detected by Flow cytometry. Data represents the mean ± SD of three independent experiments. Compared with the RAW 264.7 control group, *p<0.05; compared with the RAW 264.7+T24 control group, #p<0.05; compared with the RAW 264.7 IFN-γ group, ▲p<0.05; compared with the RAW 264.7 IFN-γ co-culture group, Δp<0.05.

To investigate whether PPS could enhance NO production in IFN-γ-stimulated macrophages, macrophages were treated with PPS (250 μg/mL). As shown in Fig 3A, the amount of NO increased significantly after treatment with 250 μg/mL PPS and reached 60 μM and 65 μM in the medium and co-culture microenvironment, respectively.

In addition, RT-PCR was used to investigate whether PPS could increase the mRNA levels of TNF-α, INOS and IL-6 in IFN-γ-stimulated macrophages. Compared with IFN-γ-stimulated cells, significantly higher INOS, TNF-α and IL-6 generation was observed in IFN-γ-stimulated cells treated with PPS (250 μg/mL) in both the medium and the co-culture microenvironment. To further investigate whether PPS could increase the secretion of TNF-α and IL-6 in IFN-γ-stimulated macrophages, RAW 264.7 cells were treated with IFN-γ (100 ng/ml) for 3 h and then incubated with PPS (250 μg/mL) for 24 h culture in medium alone or medium with T24 supernatant. As shown in Fig 3B and 3C, PPS-treated, IFN-γ-stimulated cells secreted significantly more TNF-α and IL-6 than did untreated cells. The data suggests that PPS promoted the release of NO, IL-6 and TNF-α through up-regulating the mRNA expression of INOS, IL-6 and TNF-α.

To investigate whether PPS could enhance the M1 signature marker CD86, RAW 264.7 cells were pre-incubated with 50% fresh medium or T24 cell culture supernatant for 3 h and were subsequently treated with IFN-γ (100 ng/mL) for 3 h. Then, cells treated with PPS and untreated cells were compared. Compared with the untreated cells, CD86 expression was increased by 66.0% in the treated cells. Our results showed that PPS increased CD86 expression in IFN-γ-stimulated cells, suggesting it enhanced the M1 polarization of RAW 264.7 macrophages (Fig 3E and 3F).

### NF-κB pathways are involved in IFN-γ-stimulated macrophage activation induced by PPS

To investigate whether NF-κB signaling pathways were involved in IFN-γ-stimulated macrophage activation induced by PPS, RAW 264.7 cells were pretreated with IFN-γ (100 ng/mL) for 3 h in a co-culture microenvironment. After being pretreated, the cells were cultured with PPS (250 μg/mL) for 12 h, and then flow cytometry was used to measure the surface expression of CD40 and CD284. Western blot analysis was performed to detect the levels of phosphorylated P65-NF-κB, IKB, INOS and COX-2.

**Fig 4:**
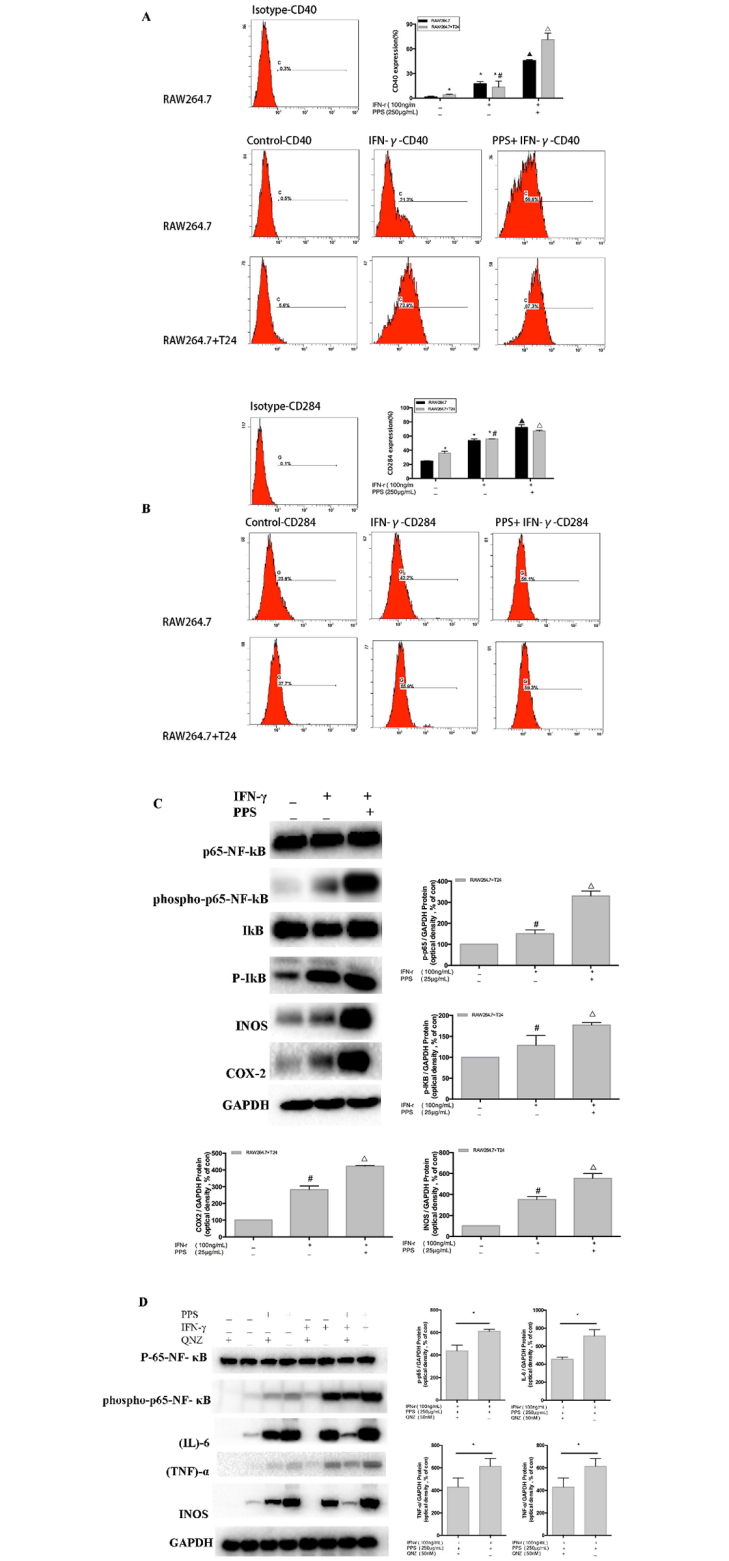
Effect of PPS on NF-κB pathways in IFN-γ-stimulated RAW 264.7 cells. (A and B) The expression of CD40 and CD284 (TLR4) in IFN-γ-stimulated RAW 264.7 macrophages treated with PPS for 24 h. Cells were collected and processed for analysis of cell cycle distribution by flow cytometry. (C) Effect of PPS (8 h) on NF-κB in IFN-γ-stimulated RAW 264.7 macrophages. phospho-P65-NF-κB, phospho-IKB, INOS, COX-2 were determined using Western blot. (D) Effect of specific NF-κB inhibitor on IFN-γ-stimulated RAW 264.7 macrophages treated with PPS for 8 h. The values of “phospho-P65-NF-κB/total P-65-NF-κB,” “phospho-IκB/total IκB,” INOS, COX-2, IL-6 and TNF-α were calculated using gray-scale values. GAPDH was used as a total protein loading control. The group without treatment with PPS was used as a control, and IFN-γ (100 ng/mL) was used as the positive control. The values are presented as the mean ± SD of three independent experiments. Compared with the RAW 264.7 control group, *p<0.05; compared with the RAW 264.7+T24 control group, #p<0.05; compared with the RAW 264.7 IFN-γ group, ▲p<0.05; compared with the RAW 264.7 IFN-γ co-culture group, Δp<0.05. Data represents the mean ± SEM of four independent experiments.

The results show that PPS increased the expression of CD40, CD284, P65-NF-κB, IKB, INOS and COX-2 (Fig 4A and 4C). In addition, PPS enhanced the activation of IFN-γ-stimulated macrophages through NF-κB pathways. IFN-γ-stimulated cells treated with PPS showed 2.0-, 1.3-, 1.4- and 1.5-fold greater production of P65-NF-κB, IκB, INOS and COX-2 protein, respectively, than that in the IFN-γ-stimulated group (Fig 4C).

### Inhibition of NF-κB pathways using a specific inhibitor

To provide further evidence that NF-κB pathways participate in IFN-γ-stimulated macrophage activation induced by PPS, PPS-treated macrophages were exposed to a specific P65-NF-κB inhibitor, QNZ, and the levels of P65-NF-κB, IΚB, TNF-α, IL-6, INOS and COX-2 inhibition were determined. As shown in Fig 4D, the NF-κB inhibitor QNZ attenuated the PPS-induced enhancement in the protein expression of P65-NF-κB, TNF-α, IL-6 and INOS by 29%, 25.2%, 37.5% and 43.8%, respectively, in IFN-γ-stimulated macrophages.

### The UV-VIS absorption of PPS

The regression equation of the standard curve was as follows: y = 0.9312x - 0.044 (y: mg/mL), R^2^ = 0.99917. In addition, the sugar content of PPS was 95.24%.

## Discussion

The immunosuppressive microenvironment is a major hurdle for treating cancer with immunotherapy, and macrophage activation is accepted as one of the most important events in immunoregulation [17]. Macrophages are characterized by their plasticity and heterogeneity, which can be functionally reprogrammed toward polarized phenotypes by exposure to cancer-related factors, stromal factors or infection [17,18]. Macrophages are the most dynamic and versatile cells involved in steady-state homeostasis, innate immune surveillance, and inflammation establishment and resolution. To elucidate how PPS facilitates the suppression of bladder cancer in vitro, it is necessary to investigate the tumor microenvironment. To some extent, RAW 264.7 cells treated with T24 cell culture supernatant may be an ideal macrophage model for in vitro studies. In our present work, we treated RAW 264.7 cells with T24 cell culture supernatant to simulate the bladder tumor microenvironment in vitro. Macrophages may be polarized into two extreme forms, i.e., the M1 or M2 phenotype, via specific signals [19]. In the tumor microenvironment, macrophages that infiltrate into the tumor are generally called TAMs, which are similar to M2 macrophages.

In general, the hallmarks of TAMs are the production of IL-10^high^, TGF-β^high^, IL-6^low^ and TNF-α^low^ [12], and they tend to facilitate chronic inflammation and promote healing, tissue remodeling and fibrosis with a regulatory and anti-inflammatory phenotype [20]. In our study, compared with the control cells, supernatant-treated RAW 264.7 macrophages released small quantities of NO, IL-6, and TNF-α and a large quantity of TGF-β, suggesting that the macrophages acquired tumor-promoting activities [21].

IFN-γ is a major macrophage activation factor responsible for M1 macrophage activation [22]. Compared with unstimulated RAW 264.7 cells treated with T24 cell culture supernatant, IFN-stimulated macrophages are characterized by the production of large quantities of inflammatory cytokines, such as IL-1β, IL-6, and TNF-α, and effector molecules, such as NO [12], in medium alone or a co-culture microenvironment. IFN-stimulated macrophages are implicated in acute inflammation and defense against microorganisms and macromolecular foreign bodies. Therefore, in this study, the treatment of RAW 264.7 cells with T24 cell culture supernatant was used as a model to simulate the bladder tumor microenvironment in vitro, and the macrophages were activated by exposure to IFN-γ.

Clinical data suggest that immunotherapy is likely to become a key part of the clinical management of cancer [17]. In addition, polyporus demonstrated clinical efficacy against bladder cancer [6,11]. Studies have found that the major active substances of polyporus are polysaccharide. Polysaccharide isolated from polyporus has potent immune-stimulating activity and anti-cancer and anti-inflammation effects both in vivo and in vitro [23]. In our previous study, PPS enhanced NO production in IFN-stimulated macrophages [24]. However, the signaling pathway underlying PPS-induced macrophage activation remains unclear. Therefore, in this study, the immune-stimulatory effect of PPS on macrophages and the mechanisms involved in PPS-induced macrophage activation were investigated.

NO, IL-6 and TNF-α are important active molecules that play key roles in the immune system. When host was threatened by exogenous pathogens, cancer or immunological diseases, these molecules are produced by activated macrophages. In our study, the concentration of NO in PPS-treated cells reached 58 μM and 64 μM in medium and the co-culture microenvironment, respectively, and was higher than that resulting from treatment with a polysaccharide from *Smilax glabra* Roxb. (500 μg/mL), as found by Zhu et al[25]. The secretion of IL-6 and TNF-α was also higher after PPS treatment than after treatment with the *Smilax glabra* Roxb. polysaccharide.

Phagocytosis activity is one of the most distinguished features of macrophage activation. It is accepted as one of the most important events in the immune response, and macrophage activation signifies the up-regulation of the innate immune response [23].

Our results show that PPS enhanced the phagocytosis activity of RAW 264.7 macrophages, implying that PPS could enhance macrophage activity to destroy threatening exogenous pathogens. As shown in Fig 2, our study showed that the phagocytosis activity was only related with PPS treatment, but not with microenvironment. CD86 is one of the most representative surface molecules of M1 macrophages [21,26–28]. In our study, PPS significantly increased the proportion of CD86-expressing macrophages among IFN-stimulated RAW 264.7 cells. This finding indicates that PPS could propel IFN-stimulated macrophages toward the M1 phenotype.

CD86 is recognized by TLR4, resulting in NF-κB activation via the myeloid differentiation factor 88 (MyD88) adapter-like (MaL)/Toll-interleukin 1 receptor domain containing adaptor protein (Tirap)-dependent pathway [29]. Pattern recognition receptors on macrophages can identify some polysaccharide to activate macrophages for participation in the immune regulation process [30]. Studies have shown that the polysaccharide krestin can bind to TLR4 to facilitate its immunomodulating effects[31].

It is reported that polysaccharide derived from *Agaricus blazei Murill* and *C*. *militaris* induces immunocyte activation through NF-κB activation [23,32]. Therefore, we attempted to demonstrate the PPS-mediated activation of NF-κB pathways in IFN-γ-stimulated RAW 264.7 cells. It is well known that NF-κB pathways play central roles in regulating immune and inflammatory processes that regulate the expression of pro-inflammatory mediators and cytokines involved in inflammation, apoptosis, and proliferation [33]. Moreover, NF-κB signaling pathway activation accounts for M1 macrophage polarization and the subsequent pro-inflammatory effects[34,35].

The phosphorylation of p65 is an essential step in the NF-κB signaling cascade, and IκB is closely associated with p65 [35]. In our study, we found that PPS increased the levels of IFN-γ-induced phosphorylated p65-NF-κB protein expression. To determine whether IκB participated in this process, we detected the cytoplasmic levels of IκB. The results show that the levels of IκB-α after IFN-γ stimulation in the presence PPS were increased. In conclusion, this study indicates that IFN-γ activated NF-κB in macrophages, and such activation was effectively determined by PPS. Meanwhile, we found that PPS increased the expression of INOS and COX-2 in IFN-γ-stimulated RAW 264.7 cells.

To provide further evidence that NF-κB pathways are involved in macrophage activation by PPS, PPS-treated macrophages were exposed to a specific NF-κB inhibitor (QNZ), and the level of inhibition of phosphor-p65-NF-κB, INOS, COX-2, IL-6 and TNF-α expression was determined. As shown in Fig 4, IFN-γ-stimulated RAW 264.7 cells treated with PPS expressed high levels of TNF-α; however, the inhibitor suppressed PPS-induced phospho-p65-NF-κB, INOS, COX-2, IL-6 and TNF-α expression.

**Fig 5:**
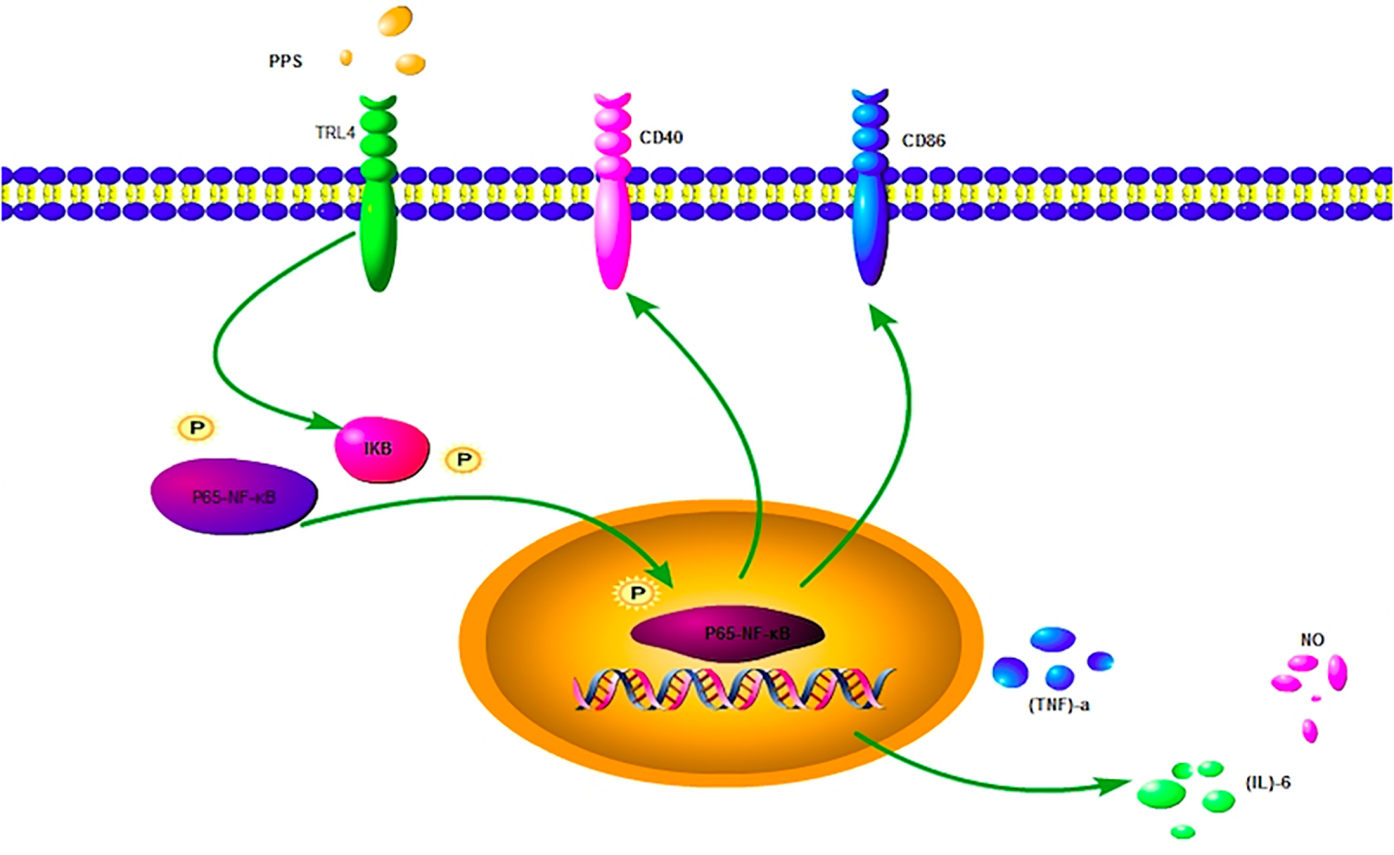
Possible molecular mechanisms of PPS effects on IFN-γ-stimulated RAW 264.7 macrophage immunomodulation.

Our findings showed that PPS could not only promote the expression of pro-inflammatory mediators via TLR4/NF-κB in IFN-γ-stimulated RAW 264.7 cells but also influence CD40 expression. The co-stimulatory molecule CD40 is expressed on antigen-presenting cells (APCs), including subsets of monocytes, macrophages, and dendritic cells [36]. In addition, CD40 is expressed in a variety of inflammatory diseases and is known to participate in the response to plant-derived polysaccharide [30]. The results of our work show that PPS markedly enhanced the expression of CD40 in IFN-γ-stimulated RAW 264.7 cells (p<0.01), indicating that PPS could strengthen the antigen-presenting capability of macrophages. As shown in Fig 5, PPS is firstly bound to TLR4, and signaling is transduced into cell to participate in the process of activating the macrophages. The process in macrophages may be as follows: the protein of p65-NF-κB and IKB are stimulated by the signal of TLR4, resulting in activation of the secretion of NO, IL-6, and TNF-α, and thereby leading to the activation of the CD 86 and CD40. Overall, the results of the present study demonstrates that PPS bounds to some surface receptors and induced an immunomodulatory response in macrophages. In addition, the possible molecular mechanisms of PPS-induced macrophage immunomodulation were found to mainly involve NF-κB pathways. While this might be the mechanism underlying the anti-cancer effects of PPS, further investigation is required.
